# Association between the *ICAM-1* gene polymorphism and coronary heart disease risk: a meta-analysis

**DOI:** 10.1042/BSR20180923

**Published:** 2019-02-22

**Authors:** De-lu Yin, Xin-hua Zhao, Yi Zhou, Ying Wang, Ping Duan, Qun-xing Li, Zheng Xiong, Yang-yang Zhang, Yu Chen, Hong He, Kai Yang, He-jian Song

**Affiliations:** Department of Cardiology, the First People’s Hospital of Lianyungang, Lianyungang 222002, China

**Keywords:** Coronary heart disease, ICAM, Meta-analysis, Polymorphism

## Abstract

Coronary heart disease (CHD) is a complex polygenic disease in which gene-environment interactions play a critical role in disease onset and progression. The Intercellular adhesion molecule 1 (*ICAM-1*) gene E469K polymorphism is one of the most commonly studied polymorphisms in this gene because of its association with CHD risks, but results were conflicting. The PubMed, Embase, and China National Knowledge Infrastructure databases were searched for case–control studies published up to November 2018. Pooled odds ratios (ORs) and 95% confidence intervals (95% CIs) were calculated to assess the association. Eleven eligible studies, comprising 3435 cases and 3199 controls, were included in the meta-analysis. The pooled result showed that the *ICAM-1* gene E469K polymorphism was significantly associated with an increased risk of CHD (OR = 1.20, 95% CI = 1.11–1.29, for the allele K versus allele E; OR = 1.66, 95% CI = 1.43–1.92, for the K allele carriers versus EE). Subgroup analysis supported the results in the Chinese populations and in the Caucasian populations. This meta-analysis suggests that the *ICAM-1* gene K469E polymorphism is associated with CHD risk and the K allele is a more significant risk factor for developing CHD amongst Chinese and Caucasians populations.

## Introduction

Coronary artery disease (CAD) continues to be a leading cause of morbidity and mortality amongst adults globally and represents a public health challenge in both industrialized and developing countries [1]. Genetic susceptibility to coronary heart disease (CHD) may be determined by specific polymorphic variants that encode proteins involved in the atherosclerotic processes. The underlying process is today believed to be predominantly mediated by cell-matrix adhesion molecules expressed on the vascular endothelium and on circulating leukocytes in response to several proinflammatory cytokines [2].

Intercellular adhesion molecule 1 (ICAM-1; human rhinovirus receptor) – a member of the large immunoglobulin superfamily – is widely expressed at a basal level and can be up-regulated by proinflammatory cytokines [2]. It is expressed on a surface of the endothelium cells, smooth muscle cells, macrophages, and activated lymphocytes. ICAM-1 plays an important role in the adhesion of circulating leukocytes to the blood vessel wall and transendothelial migration to the vascular intima [3].

A common genetic polymorphism rs5498 at position 1548 in codon 469 in exon 6 of the *ICAM-1* gene, resulting in the substitution of lysine to glutamate (K469E), might have possible functional value in the etiology of atherosclerosis [4]. The predominant homozygous allele, the heterozygous allele, and the homozygous rare allele of the ICAM-1 E469K polymorphism are known as the homozygous wild-type genotype (E/E), the heterozygote (E/K) and the homozygote (K/K), respectively. This polymorphism was suggested to affect mRNA splicing patterns that modify cell–cell interactions and influence inflammatory response [5].

A relatively large number of studies have been published on the association of ICAM-1 E469K polymorphisms and CHD risk, but the findings have been inconsistent. It is possible that a single study may have been underpowered for detection of a small effect of the polymorphisms on disease risk, especially when the sample size was relatively small. Different types of study populations and study design may also contribute to the disparate findings. To clarify the effect of the ICAM-1 E469K polymorphism on the risk for CHD, we performed a meta-analysis of all eligible case–control studies that have been published.

## Materials and methods

### Publication search

Since our study was a meta-analysis based on published articles, we did not draft a statement of patient consent or seek the approval of internal review boards. The electronic databases of PubMed, Embase, and China National Knowledge Infrastructure were searched for studies to include in the present meta-analysis, by using the terms ‘ICAM-1’, ‘intercellular adhesion molecule-1’, ‘polymorphism’, ‘CHD’ and ‘ischemic heart disease’, ‘ACS’, and ‘myocardial infarction’. An upper date limit of 25 November 2018 was applied; we used no lower date limit. The search was done without restriction on language but was limited to studies that had been conducted on human subjects. We also reviewed the Cochrane Library for relevant articles. The reference lists of reviews and retrieved articles were hand searched simultaneously. Only published studies with full text articles were included. When more than one of the same patient population was included in several publications, only the most recent or complete study was included in this meta-analysis.

### Inclusion criteria

The included studies met the following criteria: (i) evaluated ICAM-1 K469E polymorphism and CHD risk; (ii) case–control studies; (iii) supplied the number of individual genotypes in cases and controls; and (iv) indicated that the distribution of genotypes amongst controls were in Hardy–Weinberg equilibrium.

### Data extraction

Information was carefully extracted from all eligible publications independently by two authors according to the inclusion criteria listed above. Disagreement was resolved by discussion between the two authors. The following data were collected from each study: first author’s surname, year of publication, ethnicity (country of origin), total numbers of cases and controls, and numbers of cases and controls with K, E, E/E, K/E, and K/K genotypes. We did not contact the author of the primary study to request the information. Different ethnicity descent was categorized as Asian and Caucasian. We did not define any minimum number of patients as required to include a study in our meta-analysis.

### Statistical analysis

Odds ratios (OR) with 95% confidence interval (CI) were used to assess the strength of association between the ICAM-1 K469E polymorphism and CHD risk. The OR of CHD associated with ICAM-1 K469E genotype, the K allele carriers (K/E + K/K) versus E/E genotype, allele K versus allele E were calculated, respectively. Subgroup analyses were carried out with respect to ethnicity. Heterogeneity assumption was checked by the Chi-square-based Q-test [6]. A *P* value greater than 0.10 for the Q-test indicated a lack of heterogeneity amongst studies so that the pooled OR estimate of each study was calculated by the fixed-effects model (the Mantel–Haenszel method) [7]. Otherwise, the random-effects model (the DerSimonian and Laird method) was used [8]. One-way sensitivity analyses were performed to assess the stability of the results, namely, a single study in the meta-analysis was deleted each time to reflect the influence of the individual dataset on the pooled OR [9]. An estimate of potential publication bias was carried out by using the funnel plot, in which the standard error of log (OR) of each study was plotted against its log (OR). An asymmetric plot suggested a possible publication bias. Funnel plot asymmetry was assessed by the method of Egger’s linear regression test, which is a linear regression approach to measure the funnel plot asymmetry on the natural logarithm scale of the OR. The significance of the intercept was determined by the *t*-test, as suggested by Egger (*P* < 0.05 was considered representative of statistically significant publication bias) [10]. All of the calculations were performed using STATA version 10.0 (Stata Corporation, College Station, TX, U.S.A.).

## Results

### Study characteristics

A total of 14 publications [11–24], involving 3435 CHD cases and 3199 controls, met the inclusion criteria and were ultimately analyzed. Of these studies, one study [25] was excluded because the same data were available in another study [22]. Table 1 presents the main characteristics and data of these studies. Amongst the 12 publications, four were published in English and eight in Chinese. The sample sizes ranged from 150 to 1453. The diagnosis of CHD and matched controls was confirmed by coronary angiography according to the World Health Organization criteria for the confirmation of CHD.

**Table 1:** Main data of studies included in the meta-analysis

First author - year	Ethnicity (country of origin)	Number of cases/ controls	Cases					Controls				
			K	E	K/K	K/E	E/E	K	E	K/K	K/E	E/E
Shang Q-2005	Chinese	122/97	146	98	48	50	24	91	103	29	33	35
Li YJ-2010	Chinese	93/101	133	53	47	39	7	140	62	52	36	13
Lu FH-2006	Chinese	160/164	191	129	61	69	30	145	183	45	65	59
Zhang SR-2006	Chinese	173/141	274	72	111	52	10	197	85	69	59	13
Rao D-2005	Chinese	145/144	209	81	84	41	20	137	151	59	19	66
Wei YS-2006	Chinese	225/230	332	118	124	84	17	305	155	101	103	26
Zhou YL-2006	Chinese	103/197	121	85	38	45	20	266	128	102	62	33
Wang M-2005	Chinese	165/199	253	77	96	61	8	272	126	91	90	18
Jiang H-2002	Caucasian (Germany)	528/213	630	426	202	226	100	186	240	60	66	87
Milutinović A-2006	Caucasian (Slovenia)	152/215	166	138	47	72	33	239	191	65	109	41
Sarecka-Hujar B-2009	Caucasians (Poland)	191/201	142	240	12	118	61	138	268	8	122	73
Mohamed A-2010	Caucasian (Egyptian)	100/50	77	123	20	37	43	15	85	2	11	37
Luo-JY-2014	Chinese	674/779	956	392	339	278	57	1195	363	461	273	45
Yang M-2014	Chinese	604/468	861	347	305	251	48	692	244	266	160	42

Acute coronary syndrome (ACS), prior MI, stable angina pectoris cases were included. Appropriate diagnosis criteria and proper genotyping methods were used in most of the studies. There were ten groups of Asians and four groups of Caucasians. Hardy–Weinberg equilibrium had been tested for all polymorphisms in the control subjects and all were found to be in Hardy–Weinberg equilibrium.

### Meta-analysis results

**Figure 1:**
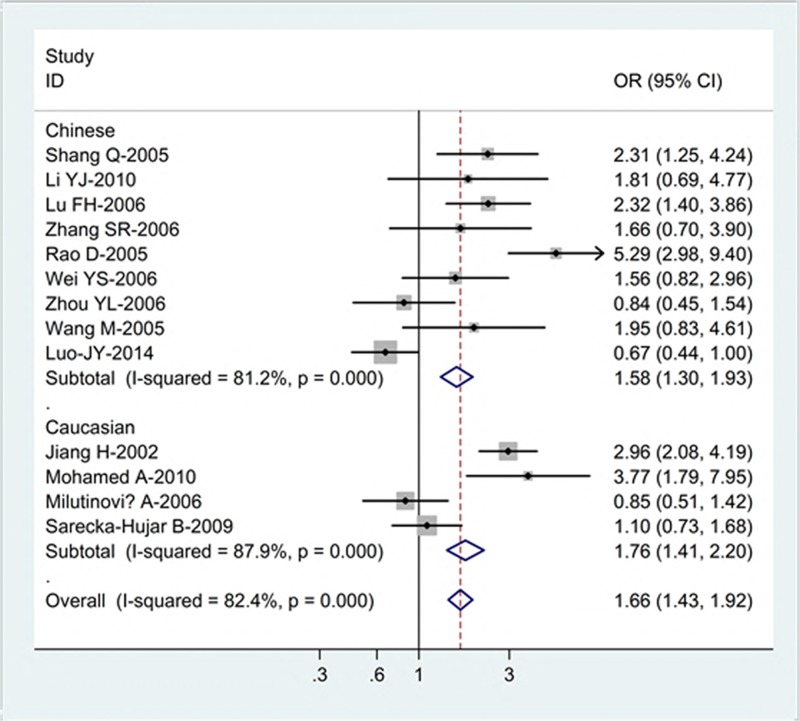
Forest plot (random-effects model) of CHD risk associated with the K469E polymorphism of ICAM-1 for the K allele carriers (K/E + K/K) versus E/E genotype Each box represents the OR point estimate, and its area is proportional to the weight of the study. The diamond (and broken line) represents the overall summary estimate, with CI represented by its width. The unbroken vertical line is set at the null value (OR = 1.0).

**Figure 2:**
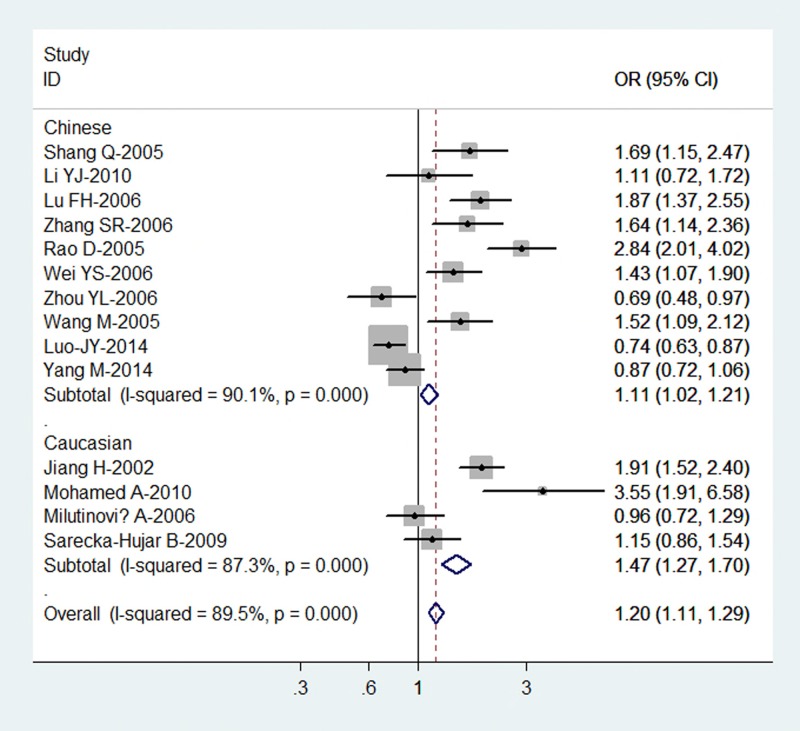
Forest plot (random-effects model) of CHD risk associated with the K469E polymorphism of ICAM-1 for the allele K versus allele E

Table 2 lists the main results of this meta-analysis. Overall, for the K allele carriers (K/E + K/K), the pooled OR for all the 14 studies was 1.66 (95% CI = 1.43–1.92; *P* = 0.000 for heterogeneity) (Figure 1), for the allele K, the pooled OR was 1.20 (95% CI = 1.11–1.29; *P* = 0.000 for heterogeneity), when compared with the homozygous wild-type genotype (E/E) (Figure 2).

**Table 2:** Main results of pooled ORs with CI in the meta-analysis

	Number of cases/controls	(KK + KE) versus EE			K versus E		
		OR (95% CI)	P (Q-test)	P	OR (95% CI)	P (Q-test)	P
Total	3435/3199	1.66 (1.43–1.92)	0.000	0.000	1.20 (1.11–1.29)	0.000	0.000
Chinese	2464/2520	1.58 (1.30–1.93)	0.004	0.000	1.11 (1.02–1.21)	0.000	0.000
Caucasians	971/679	1.76 (1.41–2.20)	0.006	0.000	1.47 (1.27–1.70)	0.000	0.000

*P*(Q-test): *P* value of Q-test for heterogeneity.

In the stratified analysis by ethnicity, significant association was identified amongst Asians for both the K allele carriers versus E/E (OR = 1.58; 95% CI = 1.30–1.93; *P* = 0.005 for heterogeneity) and allele K versus allele E (OR = 1.11; 95% CI = 1.02–1.21; *P* = 0.000 for heterogeneity) and amongst Caucasians for both the K allele carriers versus E/E (OR = 1.76; 95% CI = 1.41–2.20; *P* = 0.000 for heterogeneity) and allele K versus allele E (OR = 1.47; 95% CI = 1.27–1.70; *P* = 0.000 for heterogeneity).

### Sensitivity analyses

A single study involved in the meta-analysis was deleted each time to reflect the influence of the individual dataset on the pooled ORs, and the corresponding pooled ORs were not materially altered.

### Publication bias

**Figure 3:**
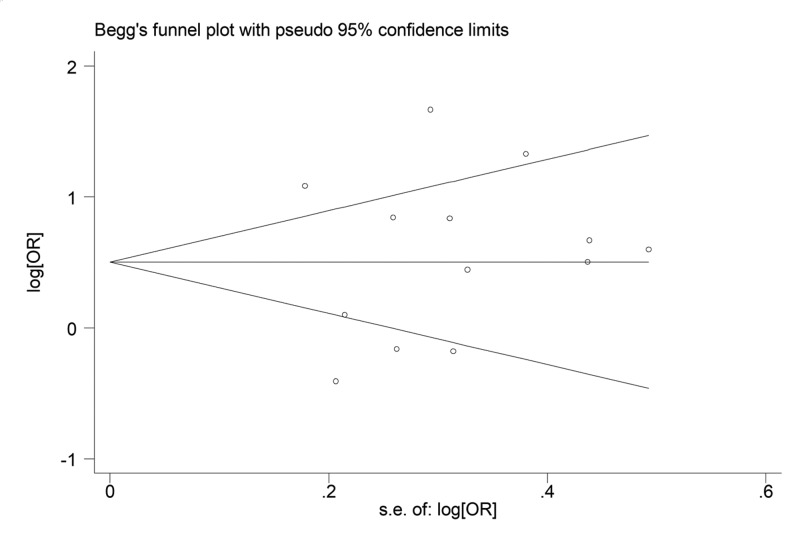
Begg’s funnel plot of the K469E polymorphism of ICAM-1 and CHD risk for the allele K versus allele

Begg’s funnel plot and Egger’s test were performed to access the publication bias of the literature. The shapes of the funnel plots did not reveal any evidence of obvious asymmetry (Figure 3). Accordingly, the Egge’s test was used to provide statistical evidence of the observed funnel plot symmetry. The results still did not suggest any evidence of publication bias (*P* = 0.45 for K allele carriers versus E/E).

## Discussion

ICAM-1 is a ligand for lymphocyte function-associated antigen 1 and integrinβ-2, making it an important player in various inflammatory/immune conditions, including atherosclerosis. Evidence implicating ICAM-1 in the pathogenesis of atherosclerosis and CHD come from both human and animal studies. Animal studies have demonstrated that transgenic mice deficient in ICAM-1 expression have reduced fatty streak formation compared with wild-type mice [26]. In 2002, Jiang et al. [20] found that the K469E polymorphism of ICAM-1 was associated with an increased risk of CHD in the Germany population. Subsequently, many investigators have sought to implicate polymorphisms of ICAM-1 in the pathogenesis of CHD. To date, the results of candidate gene case–control studies have been inconsistent. Some studies have found positive associations between the polymorphisms and CHD risk, while others have not.

Our meta-analysis summarized for the first time all the available data on the association between ICAM-1 K469E polymorphisms and CHD risk, including a total of 14 studies involving 3435 CHD cases and 3199 controls. Our results indicated that the ICAM-1 K469E polymorphisms were significantly associated with an increased risk of CHD; the K allele in the ICAM-1 K469E polymorphisms was determined to be a more significant risk factor for developing CHD in all populations. In the stratified analysis by ethnicity, significant association was identified amongst Chinese and Caucasians. However, the combined OR (1.76) for the Caucasians populations was larger than the combined OR (1.58) for the Chinese, suggesting that the K allele in the ICAM-1 K469E polymorphisms could play an important role in the pathogenesis of CHD in Caucasians. Population stratification is an area of concern and can lead to spurious evidence supporting the association between a marker and a disease, in effect suggesting a possible role of ethnic differences in genetic backgrounds and the environment they lived in. In addition, it also likely that the observed ethnic differences may be due to chance because studies with small sample size may have insufficient statistical power to detect a slight effect or may have generated a fluctuated risk estimate.

Meta-analytic methods are powerful tools for studying cumulative data from individual studies with small sample sizes and low statistical power. Pooling the effects from individual studies by a meta-analysis may increase the statistical power and can help detect modest risk differences amongst study groups. The large dataset of this pooled analysis enabled us to investigate the association between ICAM-1 E469K polymorphisms and CHD risk that could not be addressed adequately in previous case–control studies.

There are several limitations inherent to meta-analysis that should be considered when interpreting these results. First, heterogeneity is a potential problem when interpreting any results obtained by meta-analyses. We minimized this likelihood by performing a careful search for published studies, using explicit criteria for study inclusion, precise data extraction, and strict data analysis as best as we can. However, some pooled ORs were obtained from heterogeneous studies. Second, only published studies were included in this meta-analysis. The presence of publication bias indicates that non-significant or negative findings may be unpublished. At last, the studies included in the present meta-analysis are case–control studies, not randomized population-based surveys, and may be biased by problems of stratification. It is possible that controls were not recruited from exactly the same genetic population as the CHD patients.

In conclusion, the present meta-analysis suggests that the K469E polymorphism of ICAM-1 is significantly associated with an increased risk of CHD, and the K allele appeared to be a more significant risk factor for developing CHD amongst Chinese and Caucasians populations.

## Abbreviations

ACS: acute coronary syndrome
AP: angina pectoris
CAD: coronary artery disease
CHD: coronary heart disease
CI: confidence interval
ICAM1: intercellular adhesion molecule 1
OR: odds ratio
